# Genome-scale metabolic network of human carotid plaque reveals the pivotal role of glutamine/glutamate metabolism in macrophage modulating plaque inflammation and vulnerability

**DOI:** 10.1186/s12933-024-02339-3

**Published:** 2024-07-08

**Authors:** Han Jin, Cheng Zhang, Jan Nagenborg, Peter Juhasz, Adele V. Ruder, Cornelis J. J. M. Sikkink, Barend M. E. Mees, Olivia Waring, Judith C. Sluimer, Dietbert Neumann, Pieter Goossens, Marjo M. P. C. Donners, Adil Mardinoglu, Erik A. L. Biessen

**Affiliations:** 1 Central Laboratory, Tianjin Medical University General Hospital, Tianjin, China; 2 Department of Pathology, Cardiovascular Research Institute Maastricht (CARIM), Maastricht UMC+, Maastricht, the Netherlands; 3 Science for Life Laboratory (SciLifeLab), KTH-Royal Institute of Technology, Solna, Sweden; 4 PJConsulting, Natick, MA USA; 5 Zuyderland Medical Centre, Sittard-Geleen, The Netherlands; 6 Department of Surgery, Maastricht UMC+, Maastricht, the Netherlands; 7 Centre for Cardiovascular Science, University of Edinburgh, Edinburgh, Scotland; 8 Centre for Host-Microbiome Interactions, Faculty of Dentistry, Oral & Craniofacial Sciences, King’s College London, London, UK; 9 Institute for Molecular Cardiovascular Research, RWTH Aachen University, Aachen, Germany

**Keywords:** Plaque rupture, Macrophage, Genome-scale metabolic network, Atherosclerosis, Metabolomics

## Abstract

**Background:**

Metabolism is increasingly recognized as a key regulator of the function and phenotype of the primary cellular constituents of the atherosclerotic vascular wall, including endothelial cells, smooth muscle cells, and inflammatory cells. However, a comprehensive analysis of metabolic changes associated with the transition of plaque from a stable to a hemorrhaged phenotype is lacking.

**Methods:**

In this study, we integrated two large mRNA expression and protein abundance datasets (BIKE, *n* = 126; MaasHPS, *n* = 43) from human atherosclerotic carotid artery plaque to reconstruct a genome-scale metabolic network (GEM). Next, the GEM findings were linked to metabolomics data from MaasHPS, providing a comprehensive overview of metabolic changes in human plaque.

**Results:**

Our study identified significant changes in lipid, cholesterol, and inositol metabolism, along with altered lysosomal lytic activity and increased inflammatory activity, in unstable plaques with intraplaque hemorrhage (IPH+) compared to non-hemorrhaged (IPH−) plaques. Moreover, topological analysis of this network model revealed that the conversion of glutamine to glutamate and their flux between the cytoplasm and mitochondria were notably compromised in hemorrhaged plaques, with a significant reduction in overall glutamate levels in IPH+ plaques. Additionally, reduced glutamate availability was associated with an increased presence of macrophages and a pro-inflammatory phenotype in IPH+ plaques, suggesting an inflammation-prone microenvironment.

**Conclusions:**

This study is the first to establish a robust and comprehensive GEM for atherosclerotic plaque, providing a valuable resource for understanding plaque metabolism. The utility of this GEM was illustrated by its ability to reliably predict dysregulation in the cholesterol hydroxylation, inositol metabolism, and the glutamine/glutamate pathway in rupture-prone hemorrhaged plaques, a finding that may pave the way to new diagnostic or therapeutic measures.

**Supplementary Information:**

The online version contains supplementary material available at 10.1186/s12933-024-02339-3.

## Background

Ruptured atherosclerotic plaques are hallmarked by lipid accumulation, intraplaque hemorrhage (IPH), inflammation, and associated proteolysis [1]. These processes will alter local nutrient profiles and oxygen supply, with repercussions for plaque cell metabolism and functions [2]. In turn, metabolism will impact the inflammatory status of plaque cells with consequences for plaque stability [3–5].

A more precise definition of differences in cellular metabolism in stable advanced (IPH−) versus rupture-prone (IPH+) plaques will benefit not only our understanding of plaque phenotype transition but also risk stratification. So far, only a few reports aimed to map the metabolic profile of human plaque [6–8]. Though valuable, these untargeted metabolomics studies were not designed to define the (inter)cellular source of the altered metabolism [6]. Moreover, metabolomic biomarker searches generally are designed to capture dysregulated metabolites, without their metabolic context, rendering it difficult to pinpoint the underlying dysfunctional metabolic pathway.

Modeling the metabolic network in whole plaque might provide this information. Genome-scale metabolic models (GEM) [9] have been successfully applied for this purpose to dissect metabolic changes in complex diseases including non-alcoholic fatty liver disease [10, 11], type 2 diabetes [12], and Alzheimer’s disease [13], as well as in certain types of cancer, such as hepatocellular carcinoma [14], prostate cancer [15], and lung cancer [16]. The advantage of a GEM model approach is that it allows to construct a genome-scale view of plaque metabolism from plaque gene expression levels of metabolic enzymes, thereby enabling the comparison of metabolic profiles between plaque phenotypes.

Here, we constructed a human atherosclerotic plaque-specific GEM by feeding the Human Metabolic Reaction 3.0 (HMR 3.0) with transcriptomics and proteomics data of the Maastricht Human Plaque Study (MaasHPS) [17, 18] and transcriptomics data of the Biobank of Karolinska Endarterectomies (BiKE) cohort [19, 20]. Based on the plaque GEM, we dissected metabolic differences between IPH− and IPH+ plaque; these findings were then confirmed and underpinned by metabolomics data from the same MaasHPS cohort study, after which we assessed the functional implications of observed changes for disease progression by in vitro experiments.

## Methods

### Sample collection and morphology

Carotid atherosclerotic plaque tissue samples from the MaasHPS cohort (*n* = 24) were obtained from symptomatic patients who had experienced a transient ischemic attack or minor stroke resulting from carotid stenosis after which they had undergone carotid endarterectomy (CEA) surgery within 2–14 days following the onset of neurological symptoms. Detailed clinical characteristics of the patients have been previously documented and are available in Table S1 [17, 18]. These samples were sourced from the Maastricht Pathology Tissue Collection (MPTC). The carotid plaque specimens were sectioned into parallel transverse segments, each measuring 5 mm in thickness. Subsequently, every other segment was rapidly frozen in liquid nitrogen and stored at − 80 °C, while the adjacent segments were fixed in formalin for 24 h and then subjected to decalcification for 4 h before being processed and embedded in paraffin for histological analysis.

Plaques were phenotyped for stability by a pathologist (Prof. Mat Daemen) and two scientists with ample experience in human plaque phenotyping (JS and MD). Categorization of plaque stability was done at two levels: by absence (IPH−, IPH area = 0) or presence of intraplaque hemorrhage (IPH+, IPH relative area > 0) and according to the Virmani criteria for plaque staging [21]. The IPH classification was determined through computer-assisted quantitative analysis of extravascular erythrocyte deposits in Haematoxylin–Eosin-stained sections adjacent to the omics section. All IPH− plaques were staged either as pathological intimal thickening or as thick cap fibroatheroma (hence stage III–V), IPH+ plaques were stage VI (i.e., VIb), several of which showing intramural thrombi (VIc) or signs of dissection or rupture (VIa). Only CEA specimens containing both stable and unstable plaque segments were chosen for further investigation. In cases where CEA specimens were smaller, priority was given to transcriptomics and proteomics analysis, with metabolomics being conducted only if sample size permitted.

For omics analysis, samples underwent quality control checks, including assessments of RNA quality (based on RIN value > 6.0 and A260/280 ratio > 1.8; three samples did not pass the quality control). One sample exhibiting artifacts such as small surface-detached luminal fibrin clots, potentially resulting from surgery, was excluded. Additionally, samples with invalid omics profiles (one sample from transcriptomics, two samples from proteomics) were not included in the analysis. A total of 43 (16 IPH− and 27 IPH+) and 42 (16 IPH− and 26 IPH+) samples were successfully profiled for transcriptomics and proteomics, respectively. Further details can be found in Jin et al. [17].

**Fig. 1:**
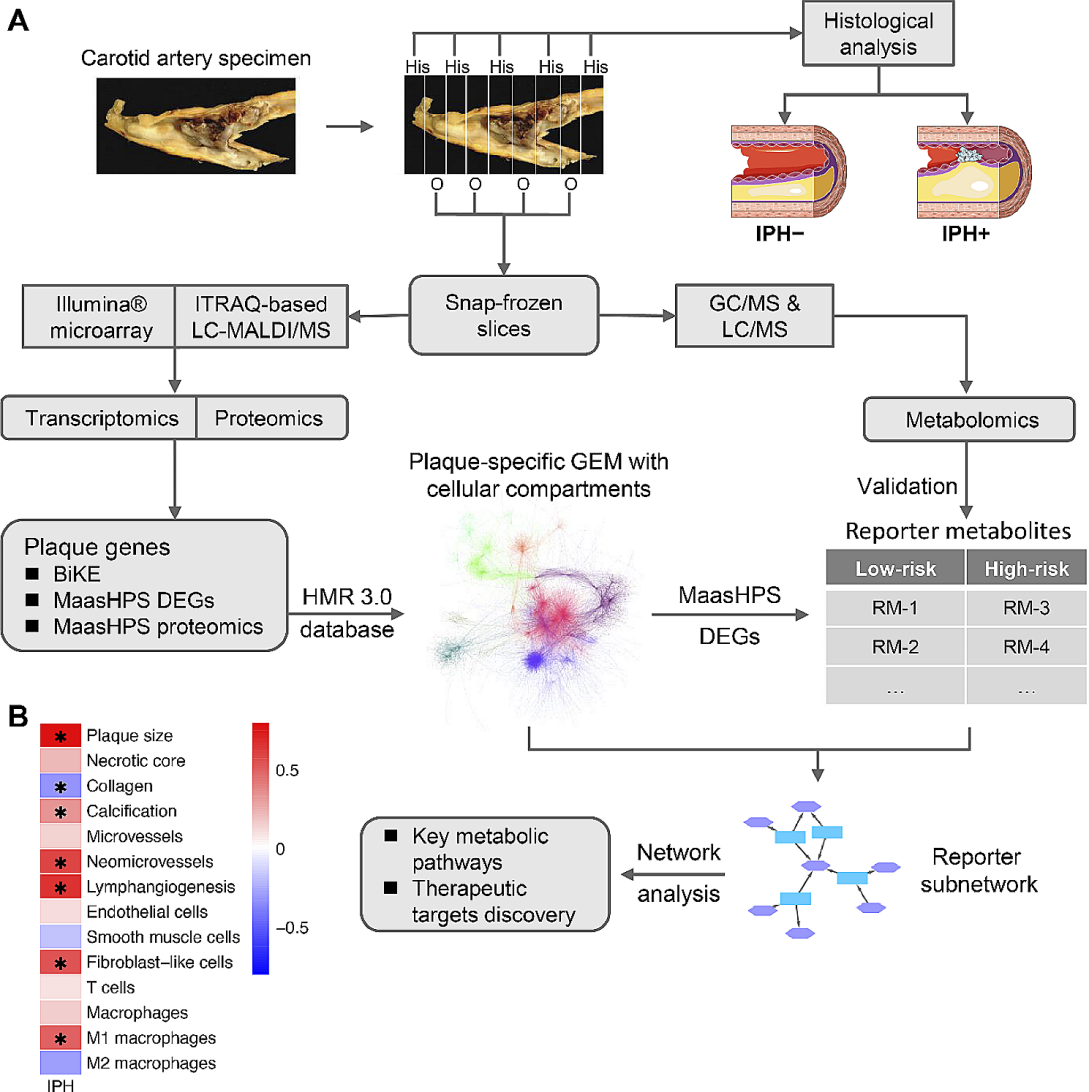
Schematic workflow. **A** The entire carotid endarterectomy specimen was cut in parallel 5 mm thick slices, snap-frozen in liquid nitrogen and stored until use. Every second slice was sectioned. After H&E staining, sections were categorized and classified histologically based on the presence/absence of IPH, a proxy of plaque stability. Sections were pulverized and aliquoted for transcriptomics, proteomics, and metabolomics analyses. Highly expressed genes in the BiKE cohort were aggregated with the MaasHPS DEGs and proteomics for building the plaque-specific GEM, after which the MaasHPS DEGs were used to infer the reporter metabolites from the well-annotated plaque-specific GEM, which were subsequently validated by the MaasHPS metabolomics. The reporter subnetwork evolved from the GEM and was used to analyze key pathways and potential therapeutic targets for plaque stability. **B** Spearman’s correlation between IPH area and other plaque traits. *P-value < 0.05

Regarding metabolomics, a total of 35 samples (9 IPH− and 26 IPH+) were successfully profiled (see below for further details). A flowchart detailing the cohort build-up is provided in Fig. 1A.

Apart from the IPH area, also plaque, media, cap, necrotic core, hemorrhage, and luminal thrombus area were quantified by morphometric analysis of H&E sections. In addition, we measured the relative area of the following histological features: CD31^+^ endothelial cells: CD31^+^ lumen-lining cells/plaque area, CD31^+^ microvessels: CD31^+^-lined structures with lumen/plaque area, CD105^+^CD31^+^ neomicrovessels (% of total plaque area), D2-40^+^ lymphangiogenesis (% of total plaque area), CD3^+^ T cell content (% of total plaque area), CD68^+^ macrophage content (% of total plaque area), iNOS^+^ (M1) macrophage content (% of CD68^+^ macrophage area), Arg1^+^ (M2) macrophage content (% of CD68^+^ macrophage area), collagen content (% Sirius red of total plaque area), αSMA^+^ smooth muscle cell (SMC) content (% of total plaque area), αSMA^−^PDGFRα^+^ fibroblast-like cell content (% of total plaque area), and calcification (% Alizarin red of total plaque area). Among these plaque traits, the level of plaque size, IPH, iNOS^+^ macrophage content, and αSMA^−^PDGFRα^+^ cell content increased, whereas Arg1^+^ macrophage decreased in IPH+ plaques [17, 18].

### Omics analysis

#### General

Each snap-frozen omics segment underwent pulverization and was then divided into aliquots for analysis across the three omics platforms. Transcriptomics and proteomics analyses utilized 5–20 mg of homogenized material each, with specific methodologies detailed in Jin et al. 2021 [17, 22]. For metabolomics analysis, both gas chromatography-mass spectrometry (GC/MS) and Liquid Chromatography with tandem mass spectrometry (LC-MS/MS) platforms were employed. The metabolomics study was conducted at TNO Quality of Life (Zeist, the Netherlands).

#### Sample preparation for metabolomics

10–20 mg aliquots of tissues were weighted and placed inside a 2-mL Eppendorf tube. The extraction of metabolites was carried out as a two-step process. Initially, polar analytes were extracted with 80%/20% methanol–water mixture used at a 1:30 w/v sample-solvent ratio. Forty-two µL of the extract was used for targeted amino acid analysis. The tissue pellet was subjected to a second extraction step with 10%/90% dichloromethane-isopropanol at a 1:30 w/v sample-solvent ratio. For the relative quantification of metabolites by both GC/MS and LC-MS/MS, quality control (QC) reference samples, representing the full metabolic diversity of the tissue, were created by pooling a total of 24 similar aliquot plaque tissue samples. Correspondingly, samples subjected to metabolomics profiling are defined as “primary samples”.

#### Metabolomics—GC/MS

GC/MS analysis was done according to the protocol of Koek et al. and Kleemann et al. [23, 24], with minor modifications. For GC/MS analysis, samples were transferred to autosampler vials and subsequently dried under nitrogen and derivatized in a two-step process. First, oximation was completed by treatment for 90 min at 40 °C with methoxamine hydrochloride in pyridine (30 µL), then trimethylsilylation was initiated by adding 100 µL of *N*-methyl-*N*-(trimethylsilyl) trifluoroacetamide (MSTFA) (50 min at 40 °C). Finally, samples were centrifuged for 20 min at 3500 rpm prior to injection. For normalization, a cocktail of internal standards (i.e., d3-leucine, d7-glucose, d3-glutamic acid, d5-phenylalanine, d4-alanine, d4-cholic acid, trifluoroacetylanthracene, difluorobiphenyl, and dicyclohexylphthalate) was added to each sample. The samples were injected using PTV injection into an Agilent (Santa Clara, CA, USA) 7890 N gas chromatograph connected to an Agilent 5975 mass spectrometer. The GC/MS analysis settings were as described in Kleemann et al. [24]. The analytical runs were arranged into three randomized batches containing a total of 35 primary and 11 QC samples. Each injection was made in duplicate. Components detected in the GC/MS runs are quantified relative to an internal standard selected individually for each component based on the smallest variability observed in the QC samples. ChemStation software (vsE02.00.493) was used for data processing. These peak area ratios were used for statistical data analysis after correction for internal standard recovery. GC/MS analytes were identified based on known fragmentation patterns of these using the TNO library of over 400 metabolites containing spectra and retention times and publicly available spectrum libraries, if opportune [24]. Metabolites with > 20% of the samples showing undetectable levels and metabolites showing a relative standard deviation of > 50% were excluded. A total of 121 peaks were processed and reported. Eleven of 121 peaks could not be identified, and the rest were mapped to 109 unique metabolites annotated by the Chemical Abstracts Service (CAS) identifier (Table S2). Batch-to-batch variations were corrected by synchronizing medians of QC samples per batch, followed by sample visualization by principal component analysis to ensure the removal of batch effects.

#### Metabolomics—LC-MS/MS

Amino acid analysis (AAA) was carried out through targeted mass spectrometric measurements using multiple reaction monitoring (MRM) scans on a 4000-QTrap instrument (MDS/ SCIEX, Concord, ON, Canada). This platform targeted 42 l-amino acids. Samples and internal standards were labeled with different isotopic variants of the 4-plex iTRAQ reagent. Ion intensities of the transitions from the protonated molecular ion to m/z 114 (standards) or to m/z 115 (samples) were monitored during the analytical runs. In a Speedvac, 40 µL of the tissue extracts were dried down. Labeling of samples and internal standards was accomplished by using the AA 45/32 starter kit (Applied Biosystems, Foster City, CA, USA). Samples were added 20 µL labeling buffer and 10 µL iTRAQ reagent coding for the m/z 115 reporter fragment. Labeling was quenched by adding 5 µL hydroxylamine to the samples. The labeled samples were dried down and re-suspended in 50 µL of the internal standard solution that contained known concentrations of pre-labeled amino acids generating the m/z 114 reporter fragment. Five µL of these samples were injected onto a C18 column on a Dionex U-3000 high-performance liquid chromatography system and resolved by a 10-minute gradient from 2% Solvent B (100% ACN, 0.1% formic acid, 0.01% heptafluorobutyric acid) to 28% B (Solvent A: 100% H_2_O, 0.1% formic acid, 0.01% heptafluorobutyric acid). Nine QC reference samples and 35 primary samples were run in a single, randomized analytical batch.

Peak integration was performed using MultiQuant (AB/Sciex). Each amino acid was quantified against its own internal standard (labeled with a different iTRAQ tag). Since the standards were present at known amounts, the relative intensities of the m/z 115 and 114 peaks were converted into absolute concentration units using the in-house pipeline (mole/mg tissue). Six of the 42 targeted amino acids were below the limit of detection. Finally, the AAA platform successfully measured 36 targeted amino acids (Table S3).

Transcriptomics data for GEM construction: The R package lumi (v2.38.0) [25] was used to analyze the MaasHPS transcriptomic data. Initially, a variance stabilizing transformation was performed, followed by normalization using the robust spline normalization (RSN) algorithm. Gene-level differential expression analysis was conducted using the Limma R package (v3.42.2) [26] with Benjamini–Hochberg correction for multiple testing. In instances where multiple transcript isoforms were present, they were mapped to the same gene utilizing the HUGO Gene Nomenclature Committee (HGNC) symbols, with preference given to the highest expressed transcript. To bolster the reliability of the GEM network construction and broaden its utility, microarray transcriptomics profiles from 126 CEA samples from the BiKE cohort were integrated into our network construction process. The BiKE cohort is a biobank collection of carotid plaque samples taken from patients with high-grade carotid stenosis undergoing CEA operation, with similar patient characteristics and omics profiling (both microarray) to the MaasHPS cohort. Details about sample collection, preservation and process can be found in previously published papers [19, 20]. Raw CEL files were downloaded from the Gene Expression Omnibus (GEO) database (accession GSE21454). To facilitate the GEM construction, data were processed by R package Affy using the robust multichip average (RMA) method, normalizing for log_2_-transformed expression values.

### Plaque-specific GEM construction

Essentially, the construction of GEM relies on the expressed enzyme-coding genes and detectable metabolic enzymes in tissues to determine all operational metabolic reactions. Therefore, to ensure the detection of lowly-expressed genes and reduction of “noisy” genes, the plaque-specific GEM was constructed utilizing expressed genes mainly inferred from the BiKE transcriptomics, since it provided a larger sample size (*n* = 126) than the MaasHPS transcriptomics (*n* = 43). Furthermore, we included significantly differentially expressed genes (DEGs, adjusted *p*-value < 0.01) and detectable proteins from the MaasHPS transcriptomics and proteomics, respectively, increasing the sampling pool to 169 plaques. In the BiKE cohort, genes with expression levels lower than the lower quartile of the full microarray expression distribution in over 80% of samples were considered not expressed, thus ensuring the retention of genes expressed in plaques while filtering out noise from transcriptomics data. This exclusion criterion was used to filter out noisy gene expression which could thwart subsequent GEM construction, while ensuring that meaningful low abundance genes expressed by the majority of plaques is retained. Furthermore, we respectively defined the genes with maximum expression higher than the upper quartile, between higher and lower quartile, and less than the lower quartile in the expressed genes as “high expression”, “medium expression” and “low expression”, respectively. A total of 4005, 8009, 4005, and 4170 genes are identified as high, medium, low, and no expression, respectively. Additionally, 4,648 strongly differentially expressed genes (adjusted *p*-value < 0.001) from transcriptomics and 803 detectable proteins from proteomics were considered “high expression” genes based on the MaasHPS cohort to forcibly include potentially important genes in the model. Our GEM construction strategy did not average the gene expression values from the two independent cohorts, which eliminated the need for batch correction or harmonization between the two transcriptomics datasets. Finally, 1486, 411, 1093, and 320 metabolic genes with high, medium, low, and no expression were used to build the plaque-specific GEM using the tINIT (task-driven Integrative Network Inference for Tissues) algorithm [27]. The final plaque-specific GEM comprised 5075 reactions, involving 3958 metabolites across 9 different cellular compartments, and encompassing 2478 enzyme-coding genes. The GEM signature was defined as the set of MaasHPS DEGs (adjusted *p*-value < 0.01) encoding enzymes participating in the reactions. Gene Ontology (GO)-based gene set overrepresentation analysis (GSOA) and gene set enrichment analysis (GSEA) were employed to analyze the GEM signature using the R packages clusterProfiler (v3.12.0) [28] with adjusted *p*-values (Benjamini–Hochberg) to indicate significance.

### Reporter metabolites and subnetwork analyses

In brief, reporter metabolite analysis aims to identify potential key hub metabolites in response to condition changes, based both on the significance of gene expression changes and on the topology of the genome-scale metabolic model [9]. All significant DEGs from the MaasHPS microarray were used to infer the reporter metabolites. In total, we identified 186, 62, and 289 reporter metabolites significantly associated with all, up-regulated, and down-regulated DEGs, respectively (*p*-value < 0.1), reflecting dysregulation of the corresponding pathways. Reporter metabolites that were marked as “up” indicated that significantly more genes of the corresponding pathway were up-regulated, and vice versa for “down”. The reporter subnetwork algorithm [9] was applied on the plaque-specific GEM to identify a connected metabolic subnetwork that is enriched with reporter metabolites. For the construction of reporter subnetwork, common metabolites, such as H_2_O, CO_2_, O_2_, H^+^, HCO_3_^−^, Na^+^, CoA, P_i_, PP_i_, AMP, ADP, ATP, NAD^+^, NADH, NADP^+^, NADPH, PAP, PAPS, FAD and FADH_2_ were removed as they associate with a multitude of metabolic pathways, and hence are not discriminative. Inclusion of these metabolites would profoundly skew the metabolic reporter towards these metabolites and hide the true structure of the subnetwork, disqualifying them for reporter subnetwork analysis [10]. Their exclusion will improve the precision of the GEM modeling without compromising detection of f.i. redox or mitochondrial metabolism.

A major reporter subnetwork consists of 373 nodes connected by 506 edges, and 101 disconnected small subnetworks (node size ranging from 3 to 33) were obtained, and only the major reporter subnetwork was selected for further analyses. Topological analysis of the reporter subnetwork was conducted using the R package *igraph* (v1.2.6) [29]. The degree (number of connected nodes of a node), closeness centrality (length of the shortest path between a node and all other nodes), and betweenness centrality (number of node-connecting shortest paths that pass through a node) were calculated for every node in the network. The reporter subnetwork was visualized by Cytoscape [30] (v3.8.0). For better visualization, extracellular matrix (ECM)-related metabolites and reactions (28 of the total 373 nodes) were removed from the reporter subnetwork.

### Plaque single-cell RNA-seq data

A publicly available plaque scRNA-seq dataset [31] consisting of 3 entire calcified atherosclerotic core (AC) plaques with 3 patient-matched proximal adjacent (PA) portions of carotid artery tissues from patients undergoing CEA, was used to explore the expression of the GEM signatures and key genes from our plaque-specific GEM in specific plaque cell populations. Expression data processed by Cell Ranger were downloaded from the GEO (GSE159677). Gene expression count values of the 6 samples were combined for pre-processing. We excluded cells that: (1) expressed less than 200 genes or more than 4000 genes; (2) had more than 10% mitochondrial counts; and (3) had less than 5% ribosomal counts. The mitochondrial genes, the MALAT1 gene, and genes expressed in less than 3 cells were excluded. In addition, we excluded 2850 cells predicted as doublets by the R package DoubletFinder (v2.0.3) [32], resulting in 41,318 cells for downstream analysis.

The scRNA-seq analyses were done by the R package Seurat (v4.1.1) [33, 34]. Expression data were normalized by the R function SCTransform, with the mitochondrial contamination and the differences between the G2M and S phase cell cycle scores regressed out. After normalization, the dimension of the data was reduced to 50 by PCA. Subsequently, the R package harmony (v0.1.0) [35] was used to remove batch effects induced by donors, whereas the differences between phenotypes AC and PA were maintained. Based on the harmony integration, uniform manifold approximation and projection (UMAP) [36] compressed the data into two dimensions for visualization.

Cells were manually annotated based on well-known markers. GEM signatures were estimated for their overall expression in plaque scRNA-seq data by AddModuleScore using the R package Seurat. In addition, several enzyme-coding genes regulating the key metabolic pathways in our reporter subnetwork were also examined based on UMAP visualization.

### Human blood cells isolation and culture

Buffy coats were collected from healthy volunteers from Uniklinik RWTH Aachen, Germany, and peripheral blood mononuclear cells (PBMCs) were isolated using ficoll-paque gradient (Sigma). Subsequently, CD14^+^ monocytes were positively selected using CD14 MicroBeads (Miltenyi), according to the manufacturer’s protocol. Monocytes were pooled from 6 to 8 donors and plated in 96-well black optical imaging plates (BD Biosciences #353219) at a density of 75,000 cells/well in RPMI1640 (Thermofisher) supplemented with 10% FCS and 1% PenStrep (Gibco) and cultured in a controlled environment (37 °C, 5% CO_2_). Monocytes were differentiated into macrophages through exposure to 100 ng/ml of recombinant human macrophage colony-stimulating factor (rh-MCSF, Immunotools) for 7 days with one medium renewal during the process.

### Stimulation of macrophages

Fully differentiated macrophages were stimulated for 24 h at 37 °C and 5% CO_2_ by one of the 28 stimuli (*n* = 8) prior to functional screening.

### Functional profiling of macrophages with silenced hub gene expression

All high content functional assays are based on fluorescent probes and cells were imaged using the high content analyzer BD Pathway 855 (BD bioscience). Nine pictures were taken per well and analyzed using Attovision image analysis software (BD bioscience) unless stated otherwise. A digital segmentation mask was created for each individual cell (region of interest, ROI). Morphological results (area, shape, granularity, and actin stress) were further processed using the CellProfiler software [37]. All other results were further processed using the DIVA software (BD bioscience). All plates contained eight control wells with unstimulated macrophages. Normalization to these plate controls was applied when plate-to-plate fluorescent intensity variation was observed. All the microscale assays were benchmarked against mesoscale counterparts. In addition, technical controls were used to validate the functional assays. Three independent experiments were performed with 6–8 replicates per condition per assay, with similar outcomes. All experiments were performed at 37 °C and 5% CO_2_ unless stated otherwise.

#### Apoptosis

To analyze the percentage of apoptosis, macrophages were incubated with 1200 nM staurosporine (Sigma) for 24 h. Subsequently, cell nuclei were stained with Hoechst 33,342 (Sigma) in complete medium. After washing with annexin binding buffer (10 mM HEPES, 140 mM NaCl, and 5 mM CaCl_2_; pH of 7.4), cells were incubated with 2.5 ng/ml Annexin-V-OG for 15 min [38]. After washing with annexin binding buffer, the plate was imaged immediately using a 10-fold objective. Mock-treated cells incubated in the absence of staurosporine served as technical control.

#### Cell shape

 To analyze cell morphology (i.e., size, shape, actin stress, and granularity), cells were first fixed with 2% paraformaldehyde (PFA) for 15 min, and subsequently stained for 30 min with Hoechst 33,342 (Sigma) and Phalloidin 594 (Santa Cruz) at room temperature. After washing with PBS, the plate was imaged using a 40-fold objective. To analyze the images, we used CellProfiler software (v3.1.9, open source) [37] to measure cell area (hypertrophy), form factor (morphology), actin stress (phalloidin 594 staining intensity and pattern), and granularity.

#### Inflammasome activation

To analyze the activation of inflammasomes, macrophages were stimulated with standard lipopolysaccharide (LPS 50 ng/ml, Invivogen) for 3 h. For negative control wells, a CRID3-inhibitor (2 µM, CRID3, Sigma) was added in the last 1 h. After washing, Nigericin (10 µM, Invivogen) was added for 1 h. Cells were transferred to ice and washed with MACS buffer (PBS, 2mM EDTA, 0.5% BSA). Subsequently, cells were incubated with FcR-block antibody (1:20, Invitrogen) for 20 min at 4 °C. Afterward, fixation buffer (PBS, 5 mM EDTA, 2% formaldehyde) was added for 20 min at 4 °C. Cells were washed twice with PBS and incubated in permeabilization buffer (PBS, 5% fetal bovine serum, 0.5% Triton-X100). Subsequently, cells were stained using anti-human ASC antibodies (1:20, Biolegend) and Fc receptor-block (1:20, Invitrogen) in permeabilization buffer over night at 4 °C. Cells were washed twice with permeabilization buffer and nuclei were stained with Hoechst 33,342 (Sigma) in PBS. The plate was imaged using a 10-fold objective.

#### Lipid uptake

 To analyze lipid uptake, fully-differentiated macrophages were incubated for 2.5 h with a mix of 8 µg/ml oxidized human Low-Density Lipoprotein (oxLDL, prepared as described before [39]) and 2 µg/ml Topfluor (Avanti Polar Lipids) in complete RPMI medium immediately after preparation. Subsequently, cell nuclei were stained with Hoechst 33,342 (Sigma) in complete medium and after washing with phosphate-buffered saline (PBS), the plate was imaged using a 10-fold objective. The negative control wells were incubated with Hoechst 33,342 only.

#### Mitochondrial stress

To analyze mitochondrial stress, macrophages were incubated with 1,200 nM staurosporine (Sigma) for 2 h. Afterward, cells were stained with 250 nM Mitotracker Red (Invivogen) and Hoechst 33,342 (Sigma) for 30 min. Stained cells were imaged using a 20-fold objective. Mock-treated cells incubated in the absence of staurosporine served as technical control.

*Phagocytosis (bead uptake)*: To analyze phagocytosis, stimulated macrophages were incubated for 1 h with 12.5 ng/ml of pHrodo-labelled Zymosan (Thermo Fisher Scientific) per well in complete medium. Subsequently, cell nuclei were stained with Hoechst 33,342 (Sigma) in complete medium and after washing with PBS, the plate was imaged using a 10-fold objective. Wells (*n* = 6 per plate) were incubated with 25 µM cytochalasin D (Sigma) for 30 min prior to bead incubation, to inhibit bead uptake, and served as technical control.

### TNF ELISA

Macrophages were treated with standard lipopolysaccharide (LPS 50 ng/ml, Invivogen) for 6 h. Subsequently, the supernatant was collected. The TNF ELISA was performed following the manufacturer’s protocol and read at 450 nm with the iMark microplate absorbance reader (Bio-Rad).

### Statistical analysis

Statistical significance of metabolite abundance between plaque phenotypes was evaluated using a two-tailed Wilcoxon rank-sum test (for non-normally distributed data) or Student’s t-test (for normally distributed data). Shapiro-Wilk test was used for the normality test. *P*-values were corrected based on the Benjamini–Hochberg procedure. Considering that the metabolomics datasets were used for validation of the key metabolites predicted by GEM, and also the moderate number of metabolites profiled, metabolites with an adjusted *p*-value < 0.1 were considered significant. One-Way ANOVA test was used for cell culture experiments. Statistical analyses were performed in R (v3.6.3) or with GraphPad Prism (v8).

## Results

### Genome-scale metabolic modeling identifies key metabolic alterations in hemorrhaged versus non-hemorrhaged human plaque

We first built a genome-scale metabolic model (GEM) for human carotid artery plaque, seeding the HMR 3.0 database, a resource for automated and semi-automated GEM reconstruction, with plaque-expressed genes, as inferred from the MaasHPS (*n* = 43; see Table S1 for baseline characteristics) [17] and BiKE microarray datasets (*n* = 126) [19, 20], as well as with MaasHPS proteomics data (protein-encoding genes; *n* = 42) using the tINIT algorithm [27] (Fig. 1A). The plaque-specific GEM contained 3958 metabolites, 5075 metabolic reactions, and 2478 enzyme-coding genes from the Human Metabolic Atlas (https://metabolicatlas.org/), with annotation of the relevant cellular compartment.

The MaasHPS cohort plaques were stratified based on the presence/absence of IPH– an established hallmark of plaque stability and event risk [40, 41]. Indeed, all IPH− plaques were phenotyped as stage III–V, whereas IPH+ plaques were stage VI. Moreover, IPH was positively correlated with plaque size and calcification (reflective of a more advanced stage), neomicrovessels, lymphangiogenesis, and M1 macrophage content (plaque inflammation), and negatively correlated with collagen content (plaque fibrosis, a feature of plaque stability), suggesting IPH as a suitable surrogate measure of plaque vulnerability (Fig. 1B). To address metabolic changes in IPH+ versus non-hemorrhaged IPH− plaque, we used all, up- and down-regulated DEGs in IPH+ versus IPH− plaques in MaasHPS as input. With the help of the Reporter Metabolite algorithm [9], we inferred reporter metabolites (i.e. metabolites likely reflective of plaque phenotype-associated changes in metabolite-relevant gene expression; see Table S4) from the plaque-specific GEM.

**Fig. 2:**
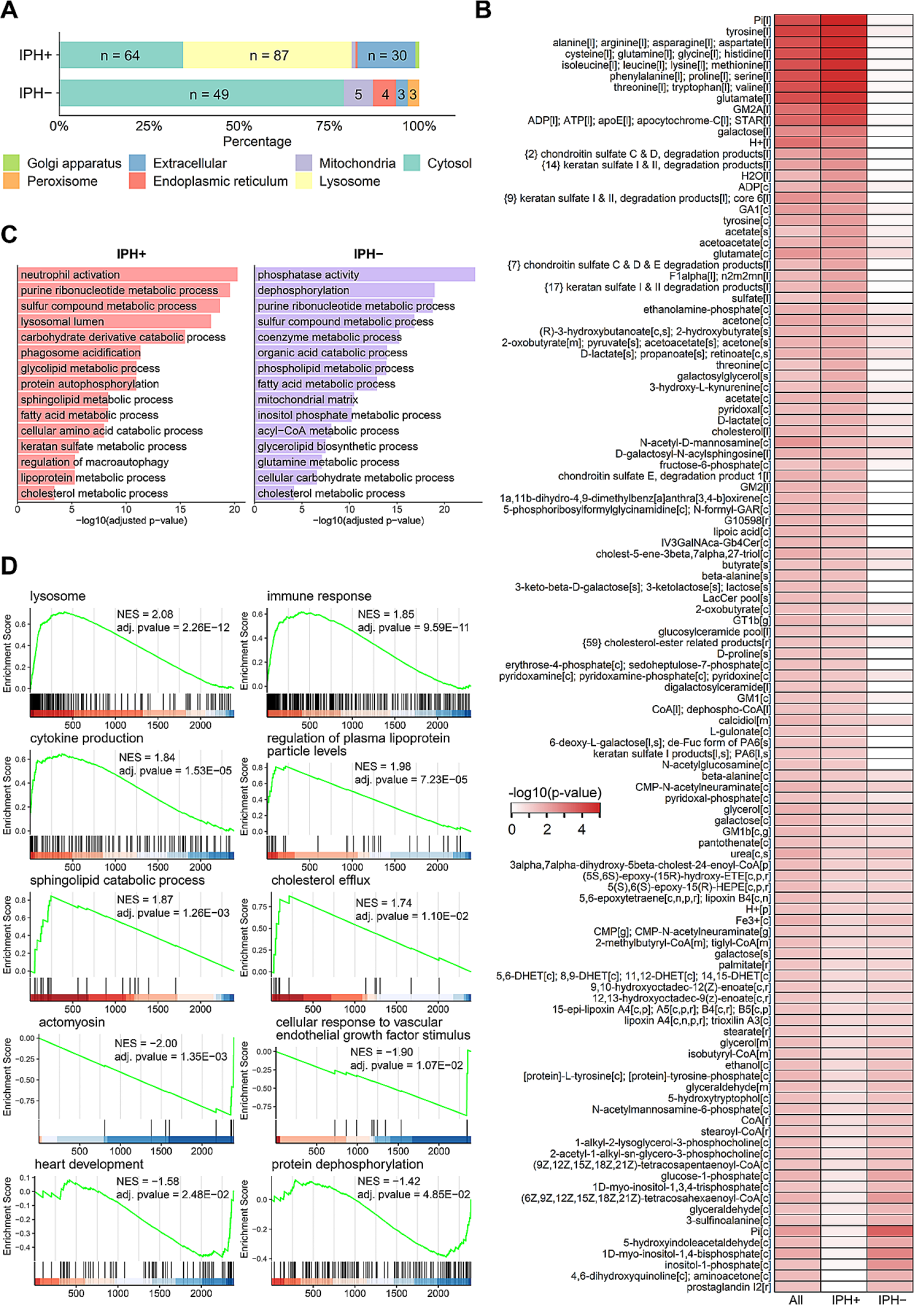
Analysis of the plaque-specific GEM network. **A** Comparison of the cellular compartment of the significant reporter metabolites inferred for IPH+ and IPH− plaques. The letter “n” indicates the number of significantly altered reporter metabolites in each cellular apartment. **B** Reporter metabolites that were significantly associated with up-/down-regulated and all genes. The number in braces shows the number of reporter metabolites belonging to the same category (denoted as “products”). The number in brackets shows the cellular compartment. **C** Gene set overrepresentation analysis of the GEM signatures. Fifteen significant GO terms were selectively shown for both up- and down-regulated GEM signatures. **D** Gene set enrichment analysis of the GEM signatures. Only the ten most significant GO terms are shown. The X-axis indicates GEM signatures sorted based on the log2 fold change (IPH+ vs. IPH−) from high to low

A first observation was that lysosome, cytosolic and extracellular metabolism was profoundly associated with IPH+ plaque, at the expense of a reduced endoplasmic reticulum (ER) and mitochondrial metabolic activity (Fig. 2A). Specifically, major changes in several lysosome-located amino acids (proteolysis), in degradation products of chondroitin- and keratan sulfate, as well as in cholesterol metabolism, could be inferred from the IPH+ versus IPH− plaque gene expression (Fig. 2B). In keeping, gene set overrepresentation analysis of the GEM signature of IPH+ plaque revealed a shift towards lysosomal, lipoprotein, and glycol/sphingolipid metabolic activity in IPH+ plaques, whereas phospholipid and mitochondrial activities were quenched (Fig. 2C). Gene set enrichment analysis of the IPH+ plaque GEM signature corroborated this finding, with significant activation of plasma lipoprotein, sphingolipid, cholesterol, and immune-related processes and lysosome compartment activity (Fig. 2D). A second pathway that our analysis predicted to be dysregulated was the inositol-pathway, as pathway related reporter metabolites were significantly “down”, meaning that the majority of pathway associated genes were down-regulated (Fig. 2B).

### Plaque metabolomics confirms GEM predicted dysregulated pathways in IPH+

**Fig. 3:**
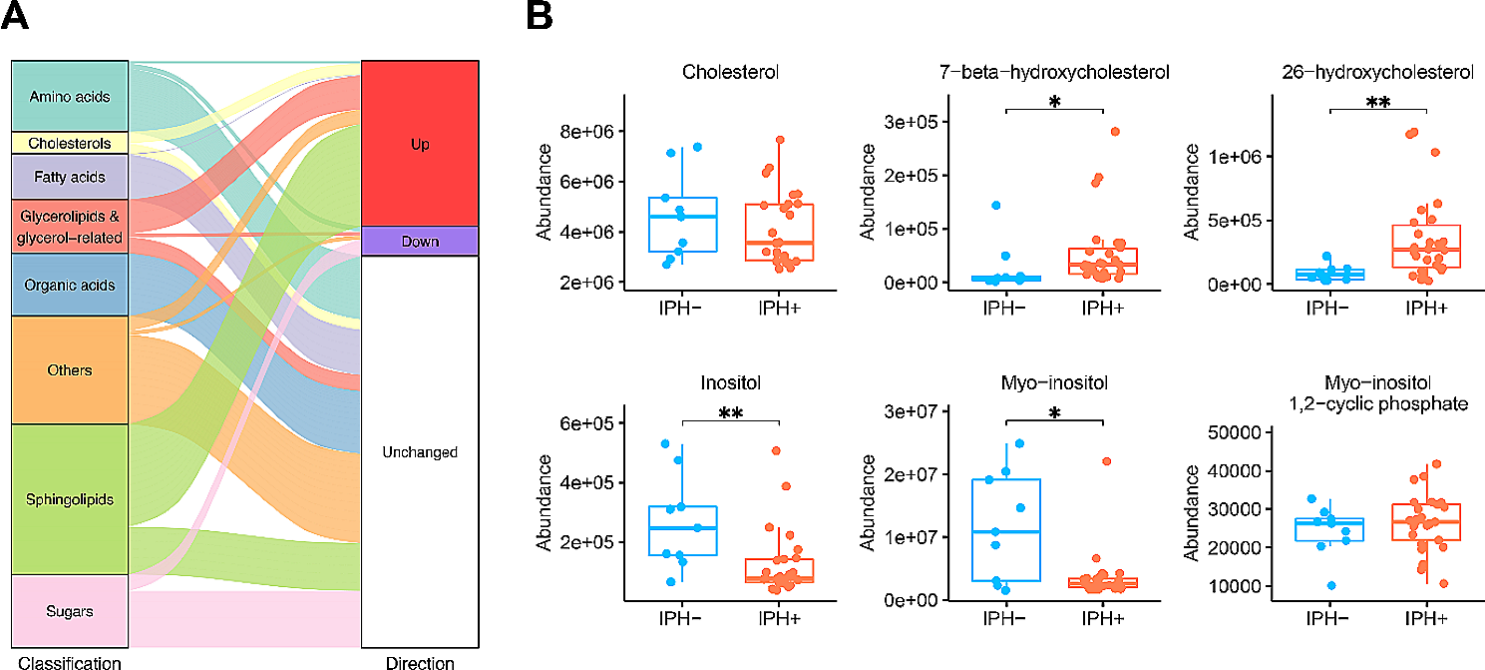
Prediction of metabolic pathway changes and their validation by metabolomics. **A** Alluvial diagram shows the categories of all the 109 detected metabolites by GC-MS, and the comparison of abundance levels between IPH− and IPH+ plaques. Up: significantly higher abundance in IPH+ plaques; down: vice versa; unchanged: no significant change between IPH− and IPH+ plaques. **B** Content of cholesterol-related and inositol-related metabolites in IPH− versus IPH+ plaques. *P*-values were adjusted by the Benjamini–Hochberg procedure. *Adjusted *P*-value < 0.1, **Adjusted *P*-value < 0.05

Next, we set out to interrogate the GEM predicted metabolic activity shifts in IPH+ plaque by metabolomics. Hereto, we evaluated the abundance of 109 metabolites in plaque GC/MS metabolomics between IPH− and IPH+ plaques (Table S2). Permutation testing, performed to estimate the random chance of finding a higher number of dysregulated metabolites from a category of metabolites (*n* = 100,000 runs), revealed that shifts in cholesterol (derivative) (2 of 3; *p*-value = 4.74E−02), sphingolipid (14 of 20; *p*-value < 1E−05), and glycerophospholipid-related metabolites (4 of 8; *p*-value = 1.90E−03) were among the most up-regulated metabolites, whereas some sugar metabolites were significantly underrepresented in IPH+ plaques (3 of 12; *p*-value = 9.54E−03) (Fig. 3A). Zooming in on cholesterol hydroxylation and inositol metabolism, we observed significant increases of 7β-hydroxy- and 26-hydroxy-cholesterol as well as decreases of inositol and myo-inositol in IPH plaque (Fig. 3B), confirming and complementing the prediction in GEM analysis.

### Identification of pivotal metabolic pathways in plaque-specific GEM

Based on the inferred reporter metabolites, we next extracted the corresponding reporter subnetwork (in other words, a connected metabolic subnetwork enriched in reporter metabolites) from the plaque-specific GEM. This reporter subnetwork was subsequently analyzed to identify the key metabolites, guided by degree centrality, closeness centrality, and betweenness centrality criteria (Table S5). For each of these topological criteria, the top 5 highest ranked metabolites were selected (Table 1), identifying cytoplasmic glutamate (Glu) and glutamine (Gln) as hub metabolites. Both metabolites were significantly associated with the genes up-regulated in IPH+ plaque (Table 1). Of note, mitochondrial glutamate, which is directly linked to cytoplasmic Glu/Gln metabolism, appeared to be a high-ranking hub metabolite as well (10th for degree and 7th for betweenness centrality).

**Table 1:** Top-ranked reporter metabolites evaluated by topological centrality

Metabolite	Rank based on network centrality			Reporter metabolite		
	Degree	Closeness	Betweenness	Compartment	Test type	*P*-value
Glutamate[c]	2	2	2	Cytosol	Up	7.45E−03
Acetate[c]	5	43	13	Cytosol	Up	1.76E−02
Glutamine[c]	12	3	1	Cytosol	Up	1.87E−02
Glycine[c]	1	28	5	Cytosol	Up	1.98E−02
Glutamate[m]	10	30	7	Mitochondria	Down	2.24E−02
NH_3_[c]	3	1	3	Cytosol	Down	9.99E−02
Serine[c]	27	5	106	Cytosol	Up	1.37E−01
AKG[c]	4	29	4	Cytosol	Up	1.46E−01
Pyruvate[c]	40	4	19	Cytosol	Up	1.85E−01

For network centrality analysis, metabolites were ranked based on a total of 238 metabolites involved in the reporter subnetwork. The test type in reporter metabolite analysis indicates if reporter metabolites was associated with up- or down-regulated genes

**Fig. 4:**
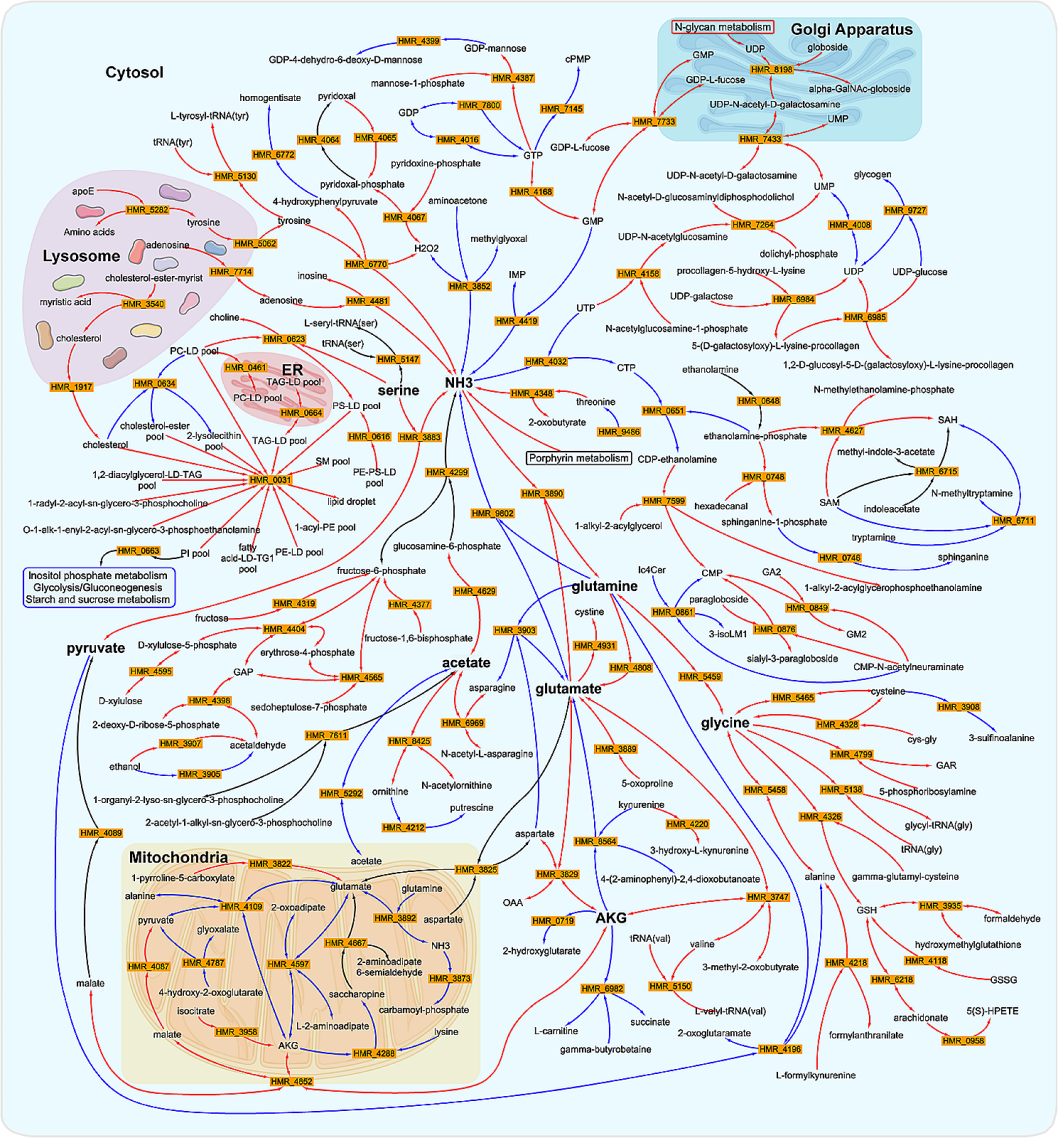
Reporter subnetwork of plaque-specific GEM

To appreciate the key metabolites in their metabolic context, we visualized the identified IPH+ plaque reporter subnetwork (Fig. 4). Cytosol Glu and Gln were connected to acetate, glycine, and several top-ranked amino acids. Metabolic reactions HMR 3890 (ATP[c] + NH_3_[c] + glutamate[c] ⇒ ADP[c] + Pi[c] + glutamine[c]) and HMR 9802 (H_2_O[c] + glutamine[c] ⇒ NH_3_[c] + glutamate[c]), which catalyze the conversion of Glu into Gln and vice versa, were the most central reactions (closeness and betweenness criteria; see Table S5). In addition, glutamate transport between mitochondria and cytosol, mediated by HMR 3825 (H^+^[c] + aspartate[m] + glutamate[c] ⇒ H^+^[m] + aspartate[c] + glutamate[m]) and implicating SLC25A13 and SLC25A12 (Table S6), ranked 17^th^ and 16^th^ among all 137 reactions based on closeness and betweenness centrality, respectively (Table S5). Thus, our findings pinpoint Glu/Gln metabolism as major, potentially dysregulated, metabolic pathway in IPH+ plaque.

Besides, we observed dysregulation of cholesterol transport from lysosomes to cytosol in IPH+ plaque (HMR 3540, H_2_O[l] + cholesterol-ester-myrist[l] ⇒ cholesterol[l] + myristic acid[l]; and HMR 1917, cholesterol[c] ⇔ cholesterol[l]), potentially reflecting the formation of intracellular lipid droplets in plaque cells (HMR 0031, see https://metabolicatlas.org/explore/Worm-GEM/gem-browser/reaction/MAR00031; and HMR 0634, PC-LD pool[c] + cholesterol[c] ⇒ 2-lysolecithin pool[c] + cholesterol-ester pool[c]). As our GEM network shows, this may alter the phosphatidylinositol pool (HMR 0663, H_2_O[c] + PI pool[c] ⇒ 1,2-diacylglycerol-LD-PI pool[c] + inositol-1-phosphate[c]), with implications for inositol phosphate metabolism, glycolysis, gluconeogenesis, and glycogen metabolism in the IPH+ plaque (Fig. 4, upper left). Overall, the reporter subnetwork findings support significant shifts in Gln/Glu and cholesterol metabolism and trafficking in unstable plaque, and provides a comprehensive and integrated view of metabolic changes in the IPH+ plaque at a sub-cellular level.

The reporter subnetwork was created from the plaque-specific GEM based on the significant reporter metabolites. Key metabolites identified through network centrality analyses, such as cytoplasmic glutamate, glutamine, serine, acetate, AKG, NH_3_, and mitochondrial glutamate in Table 1, were denoted by a large and bold font. Red arrows indicate overexpression of the associated genes for the reactions, whereas blue arrows indicate underexpression in IPH+ plaques. Black arrows indicate that the associated genes were inconsistently dysregulated. For simplicity, some metabolic pathways belonging to the same category (inositol, *N*-glycan, porphyrin) were grouped within a rectangle. Metabolic reactions with prefix “HMR” can be retrieved from the Human Metabolic Atlas (https://metabolicatlas.org/).

### Altered glutamine/glutamate pathway in plaque correlates with macrophage content

**Fig. 5:**
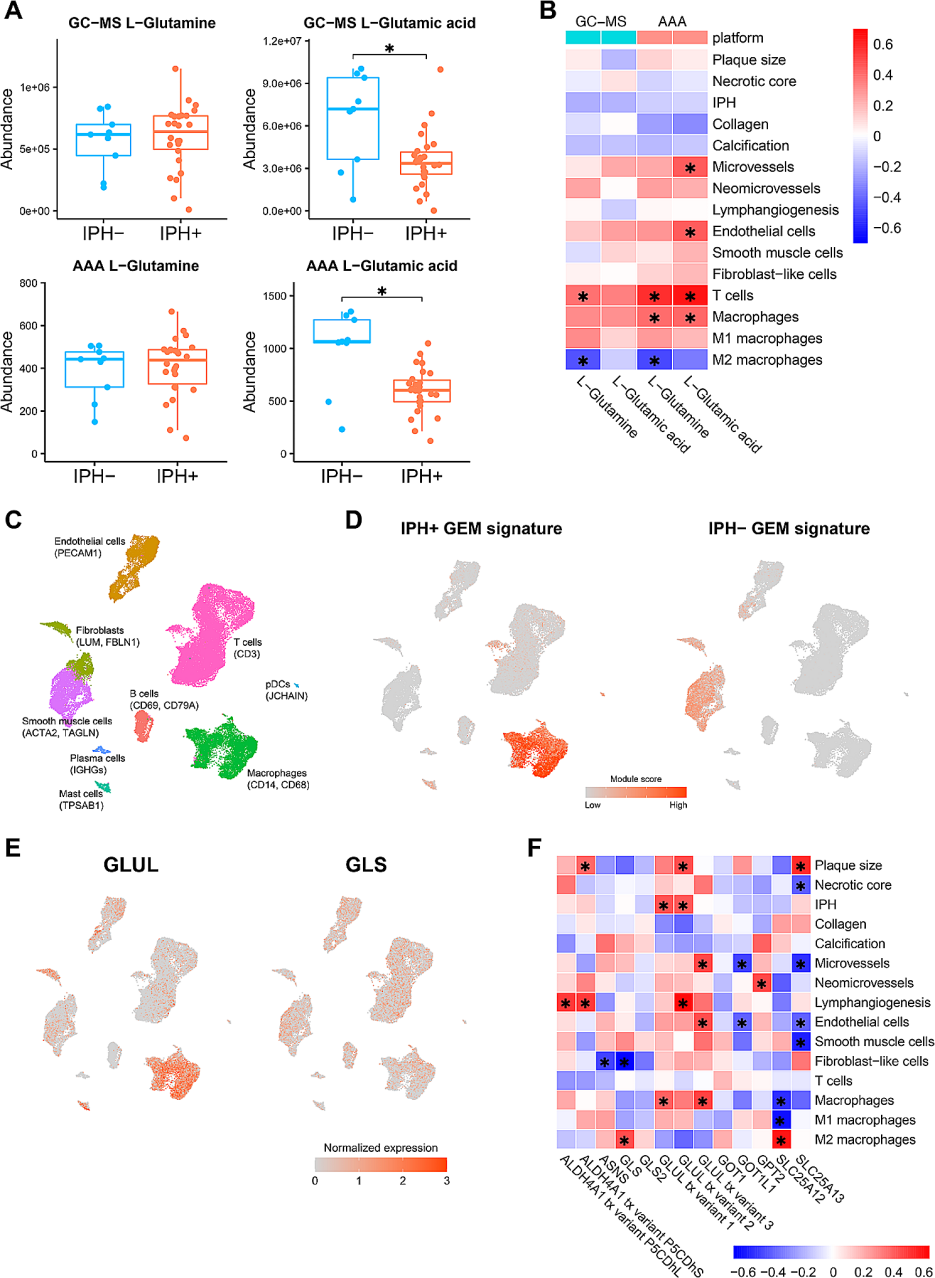
Plaque Glu/Gln metabolic pathway is associated with macrophage functions. **A** The abundance level of Glu/Gln between IPH− versus IPH+ plaques in the MaasHPS plaque metabolomics. *P*-values were adjusted by the Benjamini–Hochberg procedure. *Adjusted *P*-value < 0.1. **B** Spearman’s correlation between the metabolomic profiled abundance level of Glu/Gln and plaque traits in IPH+ plaques. *P-value < 0.05. **C** UMAP visualization showing the cell type derived from the scRNA-seq plaque dataset GSE159677. **D** The overall expression (module score) of the GEM signatures in the scRNA-seq dataset. **E** The expression level of key genes GLUL and GLS regulating Glu/Gln metabolic pathways in the scRNA-seq dataset. **F** Spearman’s correlation between the expression level of key genes for plaque Glu/Gln pathways and plaque traits in IPH+ plaques based on the MaasHPS transcriptomics dataset. *P-value < 0.05

Next, we sought to validate the in silico predicted metabolic shifts using the MaasHPS metabolomics dataset. Both GC-MS and AAA metabolomics showed a decrease in Glu (l-Glutamic acid) levels, whereas Gln levels remained unchanged in IPH+, compared with IPH− plaques (Fig. 5A). Importantly, in IPH+ plaques, Glu/Gln abundance was significantly correlated with T cell and macrophage content (Fig. 5B), linking the Gln/Glu pathway in IPH+ plaque to inflammation.

We therefore verified the GEM signatures and the expression of critical metabolic genes involved in Gln/Glu conversion and/or flux in IPH+ plaques (i.e., genes either involved in glutamate metabolism or act as transporter between cytosol and mitochondria; see Table S6) in major immune cell populations in human plaque using the scRNA-seq dataset of Alsaigh et al. (Fig. 5C) [31]. Consistent with the GSEA functional analysis of GEM signatures (Fig. 2D), we found that the IPH+ GEM signature genes were mostly expressed in plaque macrophages, whereas the IPH− signature genes were expressed in smooth muscle cells and fibroblasts (Fig. 5D), suggesting that the metabolic reactions associated with IPH+ plaques were mostly associated with plaque macrophages. Further, GLUL (responsible for Glu-to-Gln conversion, see Table S6) was highly expressed in plaque macrophages, while GLS was evenly expressed among all cell types (Fig. 5E), with the rest genes mostly underexpressed (Figure S1). This highlights the importance of GLUL over GLS in regulating Gln/Glu metabolism in plaque macrophages. Furthermore, our MaasHPS cohort data confirmed that transcript isoforms of GLUL, and Glu transporters SLC25A12 significantly correlated with plaque macrophage but not T cell content in IPH+ plaques (Fig. 5F). Of note, among the three GLUL transcript variants provided in our MaasHPS microarray data, GLUL transcript variant 2 (NCBI reference sequence: NM_001033044.1) was significantly upregulated (log_2_ fold change = 0.736, adjusted *p*-value = 3.13E−07), whereas the other two variants were not significantly dysregulated. Taken together, the prioritized Glu/Gln pathway in IPH+ plaque seems to implicate shifts in macrophage metabolism.

### Glu/Gln pathway dysregulation impacts macrophage function

**Fig. 6:**
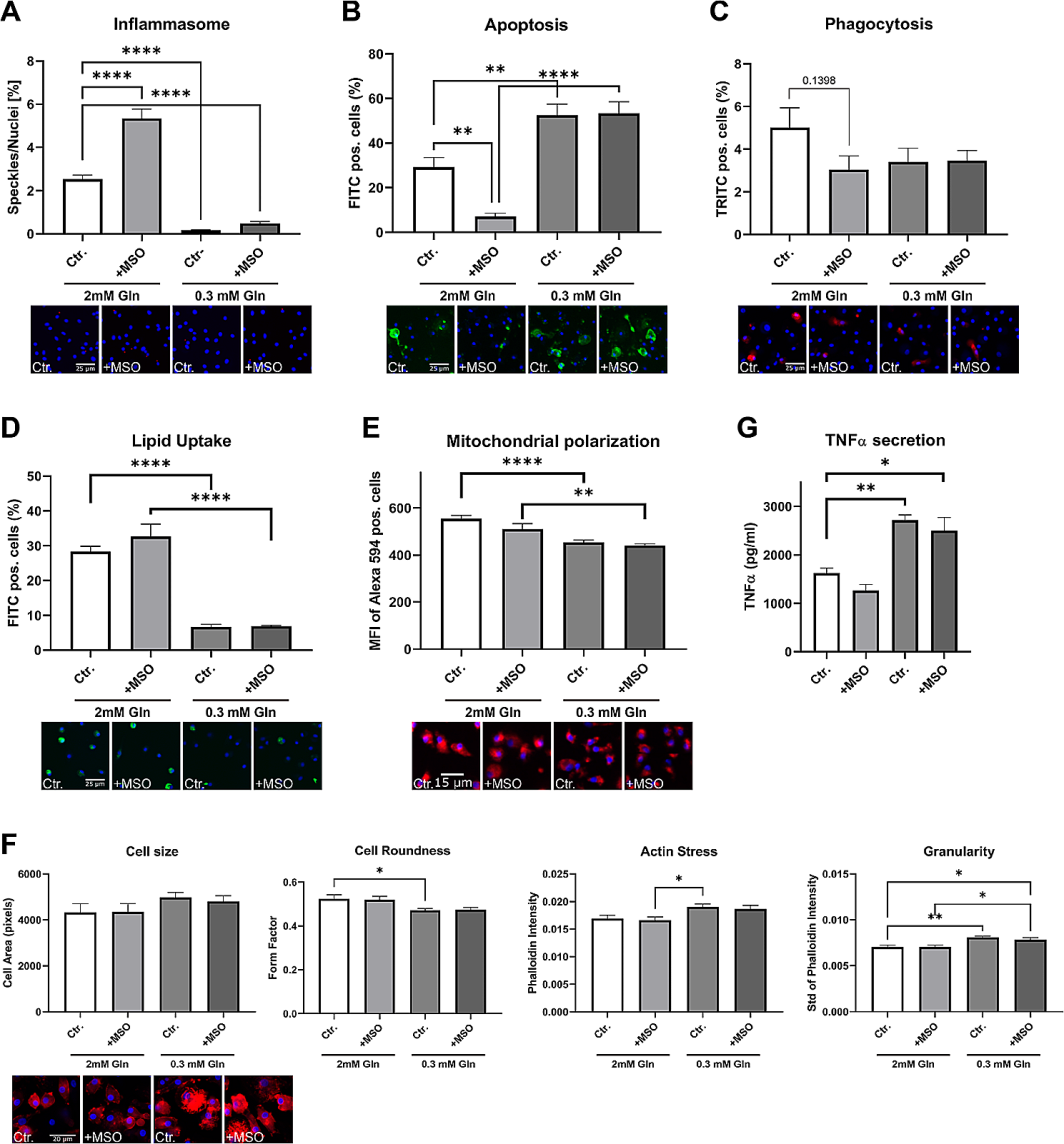
Functional changes of macrophages induced by MSO and low glutamine. Macrophages were treated with MSO (1 mM) for 24 h in the presence of indicated glutamine concentrations prior to the following functional assays: **A** Inflammasome assay was performed with LPS (50 nM) for 3 h and Nigericin (10 nM) for 1 h. Representative pictures were taken at 20x magnification. Blue = nuclei; red = ASC specks. **B** Apoptosis was measured with staurosporine (1200 nM) for 1 h. Representative pictures were taken at 10× magnification. Blue = nuclei; green = Annexin-V stain. **C** Phagocytosis assay was performed with zymosan fluorescently-labeled beads for 1 h. Representative pictures were taken at 10x magnification. Blue = nuclei; red = zymosan-labeled beads. **D** Lipid Uptake was performed with oxLDL (1 µg) for 2,5 h. Representative pictures were taken at 10× magnification. Blue = nuclei; green = fluorescently-labeled oxLDL. **E** Mitochondrial polarization was measured with staurosporine (1200 nM) for 2 h. Representative pictures were taken at 10× magnification. Blue = nuclei; red = MitoTracker Red. **F** Cell Shape was measured at baseline. Representative pictures were taken at 40× magnification. Blue = nuclei; red = phalloidin stain. **G** Macrophages were stimulated with LPS (50 ng/ml) for 6 h. *n* = 7–8 replicates for HCA experiments. *n* = 4 for TNF-ELISA. *P-value < 0.05, **P-value < 0.01, ***P-value < 0.001, ****P-value < 0.0001

Glutamine and glutamate have been shown to modulate the polarization of macrophages [42, 43]; moreover, Charvet and coworkers recently showed genetic intervention in macrophage Gln/Glu pathway has a profound effect on plaque development in mice [8]. We here mapped the effects of glutamine and glutamate pathway intervention on human macrophage functions, (i.e., apoptosis, mitochondrial stress, lipid uptake, phagocytosis, inflammasome activation, and morphology). In line with previously reported M1 polarization after GLUL inhibition, treatment of macrophages with the GLUL inhibitor methionine sulfoximine (MSO) resulted in increased LPS/Nigericin-induced inflammasome activation. In contrast, glutamine-poor medium blunted inflammasome activation, an effect that could not be rescued by MSO (Fig. 6A). Similarly, glutamine deprivation increased macrophage sensitivity to apoptosis induced by staurosporine, while MSO treatment was desensitizing (Fig. 6B). On the other hand, glutamine deprivation reduced phagocytosis and lipid uptake, but GLUL inhibition was ineffective (Fig. 6C, D). Metabolically, glutamate is an important energy source, feeding into the TCA cycle; indeed, glutamine deprivation significantly reduced the membrane potential in human macrophages (Fig. 6E), while MSO treatment again was ineffective. Furthermore, the morphology of macrophages is not affected by MSO. However, low glutamine showed a trend to increase the cell area of macrophages and significantly decrease the roundness of the cells. In addition, macrophages deprived of low glutamine exhibited higher actin stress and a higher cell granularity (Fig. 6F). Preliminary experiments showed a trend in a reduced inflammatory state of macrophages indicated by the secretion of the pro-inflammatory cytokine TNFα (Fig. 6G). While glutamine deprivation increased the secretion of the cytokine, GLUL inhibition did not affect its secretion. This requires further study to establish this finding. Collectively, the data indicate a strong effect of an altered Gln/Glu pathway and availability on atherosclerosis-related macrophage functions and phenotype in macrophages.

## Discussion

To provide deeper insights into metabolic changes in human atherosclerosis, and more specifically in the transition from stable fibrotic to unstable hemorrhaged plaque, we constructed a plaque-specific genome-scale metabolic network (GEM) by integrating two large-sized CEA plaque cohorts (BiKE and MaasHPS). The GEM covered a broad scope of metabolites and cognate reactions, enabling the prediction of key metabolic pathways associated with plaque instability. To our knowledge, this is the first GEM constructed for human atherosclerosis.

As compared to mass spectrometry-based metabolomics where pathways are inferred based on abundance level changes in a limited number of metabolites, a plaque-specific GEM offers the advantage of the higher genome-scale coverage of gene profiling. It provides a reliable high-resolution view of pathway activities at the sub-cellular level and allows us to explore plaque dysregulated metabolic pathways between cellular compartments. The network harbored many lipids, including cholesterols, sphingolipids, and glycerophospholipids, which were previously reported as major contributors to human atherosclerotic plaque inflammation and atherogenesis [44, 45].

Compared with other publicly available GEMs for cancer in the Metabolic Atlas database [46], which generally contain less than 3,000 metabolites and reactions, our plaque-specific GEM is a rather extensive model, with almost 4000 metabolites (in different compartments) linked by more than 5000 reactions. This likely reflects the complex, highly heterogeneous cellular composition of atherosclerotic plaque, harboring several subsets of T cell, macrophage, smooth muscle cell, and endothelial cell amongst others.

Applying network-based topological analyses on this plaque-specific GEM, we pinpointed the Glu/Gln pathway as a central dysregulated metabolic unit in the hemorrhaged unstable plaque. As previously reported, elevated GLUL mRNA level and local GLUL immunoreactivity are linked to macrophage M1-M2 phenotype and are associated with fibrous cap thinning, thus plaque destabilization [47]; moreover, macrophage deficiency in GLS1 to reduce macrophage glutaminolysis was seen to exacerbate atherosclerosis [8]. This is consistent with our findings. Interestingly, the GEM analysis was not suggestive of a clear Warburg effect, the aerobic glycosylation response of proinflammatory M1 macrophages. This may be due to the fact that overall the global balance in M1 and M2 subsets between stable and ruptured human atherosclerotic plaque does not notably differ, although the former phenotype may be topically enriched in the plaque shoulder [48]. In addition, the hypoxia environment in human advanced carotid plaque, and especially in unstable (IPH+) plaque may also dampen aerobic glycosylation. Whereas our study suggested local Glu deficiency within the plaque was accompanied with pro-inflammatory macrophage polarization and a vulnerable plaque phenotype, elevated plasma Glu level was reported to be associated with increased carotid intima-media thickness as well as increased inflammation [49], and with coronary heart disease, peripheral artery disease and type 2 diabetes [50].

Our analysis suggested glutamate transport between mitochondria and cytosol (HMR 3825 regulated by SLC25A12 and SLC25A13) to be causal in the Glu/Gln disparity in mitochondria versus cytosol. The changed flux into mitochondria will likely impact energy production in plaque cells such as macrophages, and thereby their functioning. Indeed, mitochondrial glutamate presence can replenish the α-ketoglutarate pool and in this way spur the TCA cycle − the immunometabolic hub of the macrophage [51]. We show that the modulation of the Gln/Glu pathway is strongly affecting macrophage functions. The inhibition of glutamine synthetase (GLUL) promotes the induction of IL-1β, however, only in the presence of glutamine via the stabilization of HIF-1α and the accumulation of succinate [52, 53]. Moreover, while GLUL inhibition reduces apoptosis in macrophages, glutamine deprivation increases their sensitivity to apoptosis. This effect has already been reported in several cell types and interestingly, is independent of energetic failure [54]. The deprivation of glutamine additionally affects macrophages’ ability to take up oxLDL and beads. Similar to reduced glutamine availability, inhibition of glutaminolysis resulted in impaired efferocytosis that requires a non-canonical glutamine pathway for effective efferocytosis for this energy-demanding task [8], suggesting a related role in phagocytosis and lipid uptake. These data provide interesting insights into Gln/Glu pathway as a promising target for immunomodulatory therapy of macrophages in atherosclerosis [55, 56], and moreover, hint at a sensitive balance between Gln and Glu. However, further research also needs to be performed on the Gln:Glu ratio and its imbalance between cytosolic and mitochondrial availability. While our plaque-specific GEM was based on transcriptomics of bulk tissue, and only hints to the cellular source of the predicted changes, an obvious next step will be to refine the analysis to single cell level, thus to allow detection of specific metabolic shifts in subsets that are relevant to disease progression.

In addition, reporter metabolites associated with glutathione (GSH) were altered in IPH+ plaques, with a significant overexpression of pathway related genes. Synthesis of GSH, a glutamate, cysteine, and glycine containing tripeptide is essential for intracellular redox control of macrophages, especially in inflammation. The impact of this dysregulation on GSH levels in plaque is hard to predict, and may also depend on cysteine levels and potentially also by cytosol glutamate levels, if severely limiting, and on the prevailing pro-oxidant conditions in plaque, increasing its consumption [57].

While pinpointing the main dysregulated metabolic processes in IPH+ plaque, our study does not allow drawing form conclusions on causality of these changes in plaque destabilization. In fact, the mere presence of extravasated erythrocytes in plaque may directly affect plaque metabolism. For instance, exposure of plaque macrophages to erythrocyte entrapped free cholesterol [58], heme and iron [59], will resonate deeply with several key metabolic pathways in the atherosclerotic plaque, including cholesterol catabolism, iron trafficking, GSH metabolism, etc. To what extent the observed metabolic changes in vesicle transport and function (degranulation, phagosome acidification and lysosomal degradation), cholesterol derivative, Glu/Gln and GSH pathways synthesis are preceding or ensuing the hemorrhage remains subject to further investigation.

Our study suggests an association of Glu/Gln pathways to macrophage presence and status in human plaque—a notion that has to be further verified in plaque single-cell expression data. However, this GEM mirrors the global metabolism in plaque, and does not provide spatial information, which considering the high spatial heterogeneity is a limitation. A second limitation is that our study leaves it unaddressed whether the identified metabolic alterations are the cause or consequence of plaque destabilization. This causality dilemma remains to be answered in future longitudinal studies. Thirdly the dichotomous sample collection and stratification did not allow for a more comprehensive monitoring of metabolic changes during plaque progression. Further study will be needed based on datasets that span the whole history of disease.

## Conclusions

In conclusion, we are the first to build a comprehensive referential GEM for human carotid atherosclerosis. Subsequent analyses of this metabolic network revealed a strong dysregulation of the Glu/Gln metabolic pathway in plaque destabilization, an effect that could be linked to macrophage presence, polarization, and functions. This GEM will be a valuable resource for the analysis of plaque metabolic studies and will benefit future efforts for immunometabolism-targeted drug design for plaque stabilizing therapy.

### Electronic supplementary material

Below is the link to the electronic supplementary material.

Supplementary Material 1.

Supplementary Material 2.

Supplementary Material 3.

Supplementary Material 4.

Supplementary Material 5.

Supplementary Material 6.

Supplementary Material 7: Figure S1. The expression level of other key genes (ALDH4A1, ASNS, GLS2, GOT1, GPT2, KYAT1, SLC25A12, SLC25A13) regulating Glu/Gln metabolic pathways in the scRNA-seq dataset.

## Data Availability

The MaasHPS and BiKE transcriptomics are publicly available in the GEO with accession number GSE163154 [https://www.ncbi.nlm.nih.gov/geo/query/acc.cgi?acc=GSE163154] and GSE21545 [https://www.ncbi.nlm.nih.gov/geo/query/acc.cgi?acc=GSE21545], respectively. The plaque scRNA-seq dataset is publicly available in the GEO with accession number GSE159677 [https://www.ncbi.nlm.nih.gov/geo/query/acc.cgi?acc=GSE159677].
