# Supplementary figures and images for: Genome-scale metabolic network of human carotid plaque reveals the pivotal role of glutamine/glutamate metabolism in macrophage modulating plaque inflammation and vulnerability
Source: Cardiovasc Diabetol. 2024 Jul 8;23:240. doi: 10.1186/s12933-024-02339-3 (PMC11232311; doi:10.1186/s12933-024-02339-3)

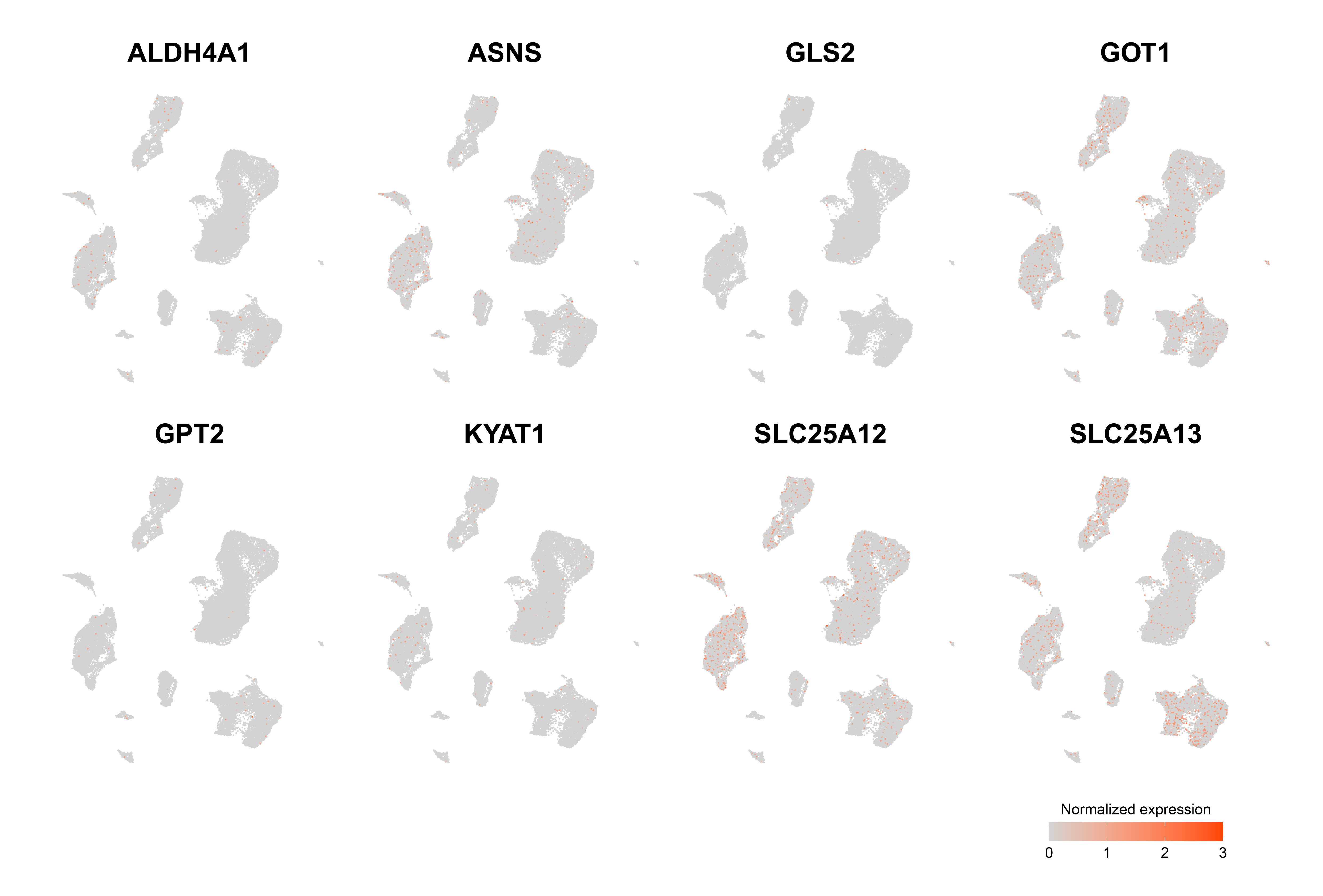

Supplement: Supplementary file 7 — Supplementary Material 7: Figure S1. The expression level of other key genes (ALDH4A1, ASNS, GLS2, GOT1, GPT2, KYAT1, SLC25A12, SLC25A13) regulating Glu/Gln metabolic pathways in the scRNA-seq dataset. [file 12933_2024_2339_MOESM7_ESM.png]
